# Protein–Chlorogenic Acid Interactions: Mechanisms, Characteristics, and Potential Food Applications

**DOI:** 10.3390/antiox13070777

**Published:** 2024-06-27

**Authors:** Mohammad Tarahi, Maryam Gharagozlou, Mehrdad Niakousari, Sara Hedayati

**Affiliations:** 1 Department of Food Science and Technology, School of Agriculture, Shiraz University, Shiraz 7144165186, Iran; tarahimohammad@yahoo.com (M.T.); niakosar@shirazu.ac.ir (M.N.); 2 Center for Organic Farming, University of Hohenheim, 70599 Stuttgart, Germany; maryam.gharagozlou@uni-hohenheim.de; 3 Nutrition Research Center, School of Nutrition and Food Sciences, Shiraz University of Medical Sciences, Shiraz 7193635899, Iran

**Keywords:** protein–phenolic acid interaction, complexes, conjugates, animal protein, plant protein

## Abstract

The interactions between proteins and chlorogenic acid (CGA) have gained significant attention in recent years, not only as a promising approach to modify the structural and techno-functional properties of proteins but also to enhance their bioactive potential in food systems. These interactions can be divided into covalent (chemical or irreversible) and non-covalent (physical or reversible) linkages. Mechanistically, CGA forms covalent bonds with nucleophilic amino acid residues of proteins by alkaline, free radical, and enzymatic approaches, leading to changes in protein structure and functionality, such as solubility, emulsification properties, and antioxidant activity. In addition, the protein–CGA complexes can be obtained by hydrogen bonds, hydrophobic and electrostatic interactions, and van der Waals forces, each offering unique advantages and outcomes. This review highlights the mechanism of these interactions and their importance in modifying the structural, functional, nutritional, and physiological attributes of animal- and plant-based proteins. Moreover, the potential applications of these protein–CGA conjugates/complexes are explored in various food systems, such as beverages, films and coatings, emulsion-based delivery systems, and so on. Overall, this literature review provides an in-depth overview of protein–CGA interactions, offering valuable insights for future research to develop novel protein-based food and non-food products with improved nutritional and functional characteristics.

## 1. Introduction

Proteins are an integral part of the human diet, as they are vital for gaining and maintaining muscle mass, renewing and repairing cells, and participating in immune responses [1]. These macronutrients consist of twenty different amino acids that are found in a wide range of foods, from meat, milk, and eggs to legumes, cereals, and oilseeds. From a techno-functional perspective, proteins also play an essential role in the food industry, serving as emulsifying, foaming, gelling, film-forming, and encapsulating agents in various food products, such as beverages, sauces, dressings, ice creams, infant formula, and baked goods [2,3]. However, they have some drawbacks in their native forms, such as low solubility and thermal stability, as well as high water vapor permeability and brittleness, which restrict their further applications. In this respect, various chemical (e.g., phosphorylation, pH-shifting, and acetylation), physical (e.g., sonication, high-pressure, and cold plasma), and biological (e.g., enzymatic and fermentation) modifications have been widely used to overcome the weaknesses of native proteins [4,5]. On the other hand, the diverse chemical structure of proteins, e.g., free amino groups, thiol groups, and tryptophan residues, facilitates covalent and non-covalent interactions with other substances, such as phenolic compounds, leading to the creation of micro- and nanoparticles with better structural, techno-functional, and nutraceutical properties [6,7].

Phenolic compounds are synthesized as secondary metabolites in higher plants through the shikimate, pentose phosphate, or phenylpropanoid pathways in order to defend against UV radiation, pathogens, parasites, and predators. They consist of aromatic rings with hydroxyl substituents, ranging from simple to highly polymerized structures. These compounds, abundant in the human diet, act as natural antioxidants, colorants, and preservatives in various food products [8,9,10]. Chlorogenic acids (CGAs) are esters of quinic acid and one or more caffeic, ferulic, p-coumaric, and sinapic acids, which belong to hydroxycinnamic acids, a subgroup of phenolic compounds with a C6–C3 skeleton [11]. CGAs consist of a wide range of isomers, particularly 3-caffeoylquinic acid (3-CQA), 4-caffeoylquinic acid (4-CQA), and 5-caffeoylquinic acid (5-CQA), found in coffee, tea, fruits, and vegetables. Among them, 5-CQA is known as the main proportion of CGA isomers in the human diet and is also the first CGA to be commercialized [12]. CGAs are recognized for their excellent biological benefits, such as high antioxidant, anti-inflammatory, anticarcinogenic, and antimicrobial activities. These health-promoting effects are mainly attributed to their ability to donate electrons, which helps delay or prevent oxidative damage caused by reactive oxygen species (ROS) or pro-oxidant metal ions. On the other hand, CGAs are also well known for their antihypertensive effects and positive impact on insulin action, glucose tolerance, and lipid metabolism [13,14].

In recent years, the complexation/conjugation of proteins with CGAs has gained much attention not only as a sustainable method to modify protein functionalities but also as a strategy to design innovative bioparticles with desired bioactive properties in the pharmaceutical and food sectors [15]. Mechanistically, the protein–CGA interactions can be divided into two main groups, namely, covalent (chemical or irreversible) and non-covalent (physical or reversible) interactions. The conjugation of proteins and CGAs can occur through alkaline, free radical, or enzymic methods. In this regard, CGAs are oxidized to reactive quinones, which subsequently react with free amino groups or sulfhydryl groups of proteins, resulting in the formation of covalent linkages. On the other hand, non-covalent interactions, such as hydrogen bonds, hydrophobic and electrostatic interactions, and van der Waals forces play a crucial role in forming protein–CGA complexes, depending on the relative number of negatively and positively charged residues on the protein, as well as the pH and concentration of electronegative atoms (e.g., N, O, or S) in the medium [16,17]. In general, covalently linked protein–CGA conjugates are stronger and more stable than non-covalent protein–CGA complexes as they involve the sharing of electron pairs, although both covalent and non-covalent interactions can alter the properties of proteins [8].

The interactions between proteins and CGAs are influenced by several factors, including the composition, molecular weight, charge, and type of proteins; the concentration of CGAs; the type and number of binding sites on both proteins and CGAs; and the pH and temperature of the environment [2,14]. These interactions may change the structure of proteins, which can affect their techno-functional properties, such as solubility, digestibility, and thermal stability, as well as their emulsifying and foaming capacity. Additionally, the nutritional properties of proteins, e.g., antioxidant activity, can be affected by CGAs. Therefore, this review aims to provide an in-depth understanding of protein–CGA conjugates and complexes under different conditions in order to improve the functionality of animal- and plant-based proteins in various food systems.

## 2. An Overview of CGA

### 2.1. History, Chemistry, and Resources

Historically, studies on CGAs can be traced back to the study of Robiquet and Bourton in 1837, as highlighted by Lu et al. [18]. However, the first documented reference dates back to 1844 by Rochleder, who isolated an acidic substance from green coffee beans that could be precipitated as a salt. Two years later in 1846, the same researcher suggested the empirical formula of C16H9O8 for this free acid, which is yellow in ammonia solution and turns green when exposed to oxygen. That is why this compound is called “chloro” despite the absence of chlorine. Later that year, the term “CGA” was first mentioned by Payen, who isolated a crystalline caffeine-potassium chlorogenate with an empirical formula of C14H8O7 from Coffea arabica L. beans. About half a century later, in 1907, pure white crystals with a melting point of ~207 °C were obtained from the alkaline hydrolysis of quinic acid and caffeic acid, and a year later, Gorter proposed the empirical formula of C32H38O19 for these crystals. To make these observations consistent with the proposed empirical formula, he also suggested that quinic acid combines with caffeic acid to form hemi-CGA [19,20]. Later, the process of gradual discovery continued, and in 1920, Freudenberg reported the hydrolysis of CGA by the enzyme tannase, which released equimolar amounts of quinic acid and caffeic acid. Fischer and Dangschat (1932) finally deduced that CGA is 3-caffeoylquinic acid (3-CQA), which is known as 5-O-caffeoylquinic acid (5-CQA) under the current recommendations of the International Union of Pure and Applied Chemistry (IUPAC). Moreover, further developments, including the isolation of cynarin (now known as 1,3-O-dicaffeoylquinic acid (1,3-diCQA)) from artichoke leaves, were reported by Panizzi and Scarpati in 1954, marking the expansion of CGA derivatives beyond its initial identification [21].

Since the mid-1990s, with the advances in analytical techniques, including nuclear magnetic resonance (NMR) spectroscopy and high-performance liquid chromatography (HPLC), more than 400 isomers of CGA have been identified from various sources. CGAs constitute a large family of natural esters formed between quinic acid and one or more trans-hydroxycinnamic acid derivatives, including caffeic, ferulic, p-coumaric, and sinapic acids [22]. Considering the identity, position, and number of acyl residues in these compounds, CGAs can be divided into four main categories: (1) mono-esters of caffeic acid (i.e., caffeoylquinic acids (CQAs)), ferulic acid (i.e., feruloylquinic acids (FQAs)), and p-coumaric acid (i.e., p-coumaroylquinic acids (pCoQAs)); (2) di-esters, tri-esters, and tetra-esters of caffeic acid (i.e., diCQAs, triCQAs, and tetraCQAs, respectively); (3) mixed di-esters of caffeic and sinapic acids (i.e., caffeoylsinapoylquinic acids (CSiQAs)) or caffeic and ferulic acids (i.e., caffeoylferuloylquinic acids (CFQAs)); and (4) mixed esters of caffeic acid residues with dibasic aliphatic acid residues, such as succinic, oxalic, and glutaric [23]. Currently, it is clear that using the term “CGA” for a single chemical structure is wrong. However, 5-CQA is the main existing chemical form and the highest proportion of CGA isomers in nature (70–80% of the total CGA), so CGA is generally known as 5-CQA internationally [12,24].

**Figure 1:**
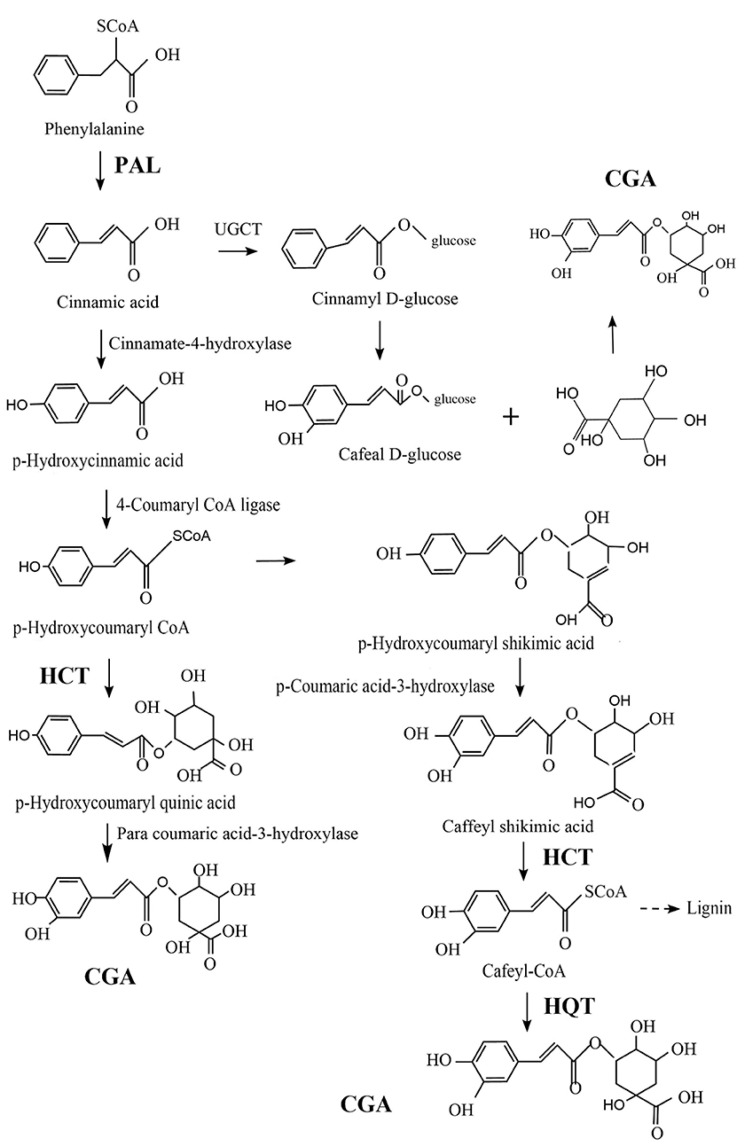
Three possible pathways for the biosynthesis of chlorogenic acid (CGA), adapted from Wang et al. [25]. HCT: quinic acid hydroxyl cinnamyl transferase; HQT: quinic acid cinnamate hydroxyl transferase; and PAL: phenylalanine-ammonia-lyase.

CGA is one of the most abundant polyphenols in the human diet, which can be found in a wide range of food products, from fruits and vegetables to spices and beverages. As shown in Figure 1, there are three possible pathways for the biosynthesis of CGA (or 5-CQA) as a secondary metabolite of certain plants. Generally, CGA is formed from phenylalanine through the shikimic acid pathway under the action of phenylalanine-ammonia-lyase (PAL), quinic acid hydroxyl cinnamyl transferase (HCT), and quinic acid cinnamate hydroxyl transferase (HQT) enzymes in the process of plant aerobic respiration. In this process, PAL plays an essential role by catalyzing the dissociation of ammonia from phenylalanine to generate cinnamic acid. Furthermore, in acylation, another crucial step in secondary metabolite generation, catalyzed by HCT, p-hydroxycoumaryl-CoA provides acyl to quinic or shikimic acids in order to form p-coumaroyl-quinic or -shikimic acids, which is the substrate of hydroxylated cinnamyl transferase (C3H). HCT can also eliminate shikimic acid from the C3H-catalyzed product, converting it into Cafeyl-CoA. On the other hand, HQT has acyl acceptor specificity that can catalyze the last step of CGA biosynthesis, by catalyzing the transesterification of quinic acid and Cafeyl-CoA [25,26,27]. These three pathways may exist simultaneously in plants, but there is a primary and secondary relationship between them that may be related to plant type and external environmental conditions, such as oxygen and light [28].

The main dietary sources of CGAs include coffee beans, apples, cherries, berries, apricots, plums, peaches, carrots, potato tubers, tomatoes, eggplants, artichoke leaves, sunflower seed kernels, tea leaves, and so on. Among them, the highest levels of CGAs have been reported in coffee beans, ranging from 3.4 to 4.8% (dmb) for Coffea canephora P. and 7.88 to 14.4% (dmb) for Coffea arabica L. In addition to different total CGA levels, different isomers of CGA, such as CQAs, FQAs, pCoQAs, and their mixed di-esters, can be isolated from coffee beans [29,30]. Furthermore, Mills et al. [31] reported different amounts of 5-CQA for fresh ground coffee (8.19–23.78 mg/200 mL) and instant coffee (9.45–41.05 mg/200 mL), which indicates the possible isomerization or degradation of CGA compounds during coffee processing. In a comprehensive review by Lu et al. [18], the content of 5-CQA in various dietary sources has been investigated, ranging from 1.4 to 28.0 mg/g in eggplant, 0.3 to 18.8 mg/g in carrot, 0.02 to 3.07 mg/g in potato, 0.8 to 2.1 mg/g in pear, 0.2 to 2.1 mg/g in artichoke, 0.4 to 1.2 mg/g in apple, 0.7 to 0.9 mg/g in pepper, and 0.19 to 0.38 mg/g in tomato. Therefore, the qualitative and quantitative characteristics of CGA compositions may vary not only due to the plant parts (e.g., leaves, seeds, roots, tubers, and flowers) and varieties but also due to the plant physiological stages, storage conditions, and processing methods.

### 2.2. Biological Properties

**Figure 2:**
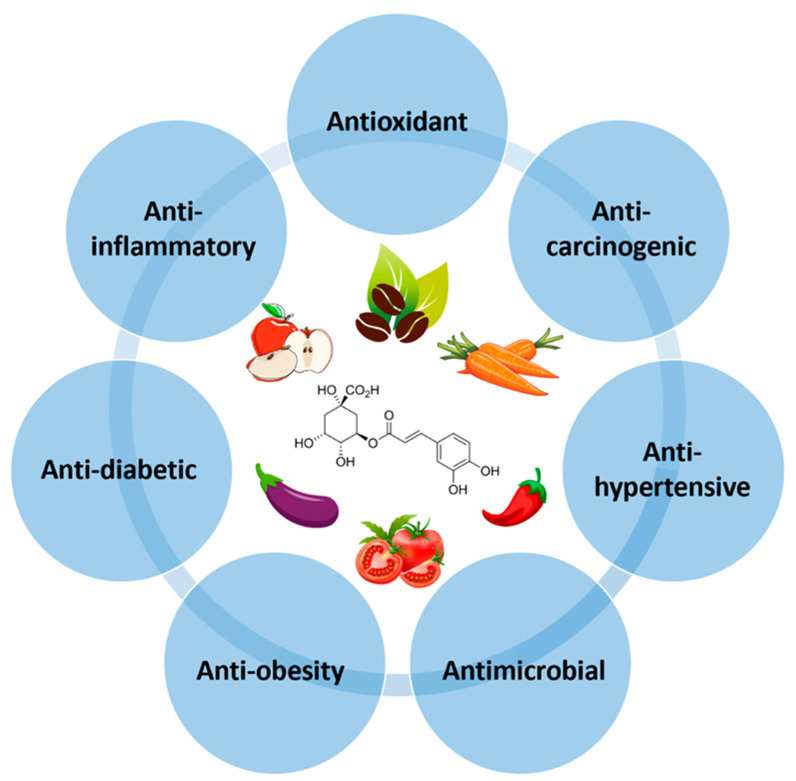
Biological properties of chlorogenic acids (CGAs).

CGA, a secondary metabolite of higher plants, has gained a lot of attention for its numerous health benefits, including antioxidant, anti-inflammatory, anticarcinogenic, antihypertensive, antidiabetic, anti-obesity, and antimicrobial activities (Figure 2). These health-promoting effects of CGAs are mainly attributed to their ability to combat oxidative stress by scavenging free radicals, acting as a metal chelator, reducing lipid peroxidation, and inhibiting NADPH oxidase activity [32].

**Figure 3:**
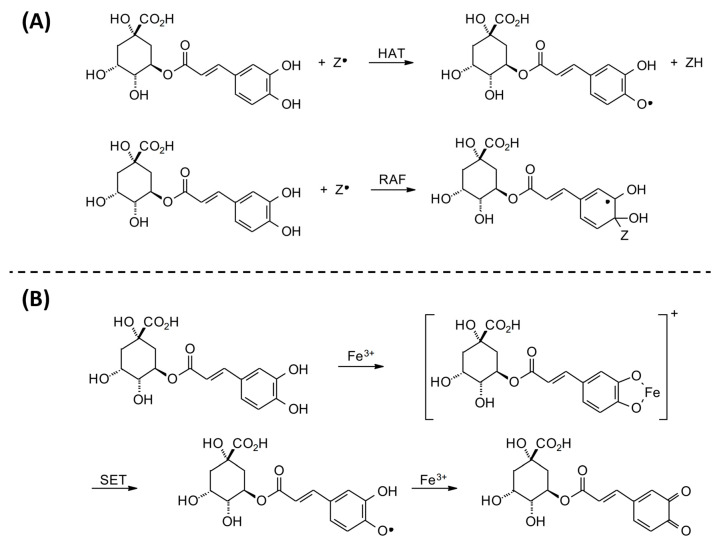
Reaction mechanisms for (**A**) possible radical scavenging activity and (**B**) iron-chelating ability of chlorogenic acid (CGA).

The production of free radicals is associated with the routine physiological activities of the human body. Oxidative stress is caused by the imbalance between ROS and the antioxidant defense system, which may cause a series of abnormal changes in the body [33]. The radical scavenging ability of CGAs can be explained by two possible mechanisms: (1) the mechanism of hydrogen atom transfer (HAT), in which a free radical removes a hydrogen atom from CGAs; or (2) the radical adduct formation (RAF) reaction, in which the free radical is added to CGAs in order to form an intermediate radical (Figure 3A). In addition, CGAs can interact with redox-active transition metals, specifically forming a chelate with Fe (III) and subsequent single-electron transfer (SET) to produce semiquinone and Fe (II), which then reacts with another Fe (III) and produces the corresponding quinone (Figure 3B) [34]. On the other hand, ROS are also identified as a major upstream factor in the signaling cascade involved in inflammatory responses. In this regard, CGAs can reduce the risk of several chronic diseases, such as diabetes, cancers, and cardiovascular diseases, by inhibiting inflammatory mediators like TNF-α, IL-6, and IL-1β [35]. In addition, CGAs can act directly on the NF-κB signaling pathway to control the expression of anti-inflammatory and pro-inflammatory factors. In this respect, some studies have shown that CGAs block the NF-κB pathway by downregulating CD14 and p65 and preventing phospho-p65 from entering the cell nucleus, which is very effective in maintaining the integrity of the cell membrane and inhibits the secretion of pro-inflammatory cytokines by reducing CD14 to block the NF-κB pathway [36,37]. These findings demonstrate the potential of CGAs to treat various chronic diseases, such as breast cancer [38]. In a review study, Nwafor et al. [39] also reported the anticarcinogenic effects of CGAs against other types of cancers, such as lung, colon, liver, blood, brain, bone, skin, kidney, and pancreatic cancers.

Moreover, it has been proven that CGA has positive effects on obesity and diabetes by affecting critical transcription factors and enzymes regulating lipid and glucose metabolisms. In this regard, Cho et al. [40] discovered that CGA supplementation in mice with diet-induced obesity significantly inhibited fatty acid synthase (FAS), enhanced fatty acid β-oxidation activity, and increased peroxisome proliferator-activated receptor α (PPARα) expression when compared to the control group. Additionally, Huang et al. [41] demonstrated that CGA reduces lipid synthesis by altering the expression of PPARα and liver X receptor α (LXRα), which are involved in multiple intracellular signaling pathways. Furthermore, it has been suggested that CGA can decrease the expression of hepatic glucose-6-phosphatase and increase adiponectin receptors, adiponectin, and the phosphorylation of AMP-activated protein kinase (AMPK) in late diabetic mice [42]. These changes have been associated with improvements in fasting glucose, glycosylated hemoglobin, triglycerides, cholesterol, hepatic steatosis reduction, increased insulin sensitivity, and improved glucose tolerance [11,43]. CGA is also known for its potential as an antihypertensive agent. For instance, in a randomized clinical trial with 117 hypertensive subjects by Kozuma et al. [44], CGA derived from green coffee extract was shown to effectively lower blood pressure without any side effects. In further studies, the results demonstrated that CGA can reduce blood pressure in spontaneously hypertensive rats (SHRs) through various mechanisms, including increasing nitric oxide (NO) bioavailability and inhibiting vascular NADPH oxidase activity [45,46]. Human trials also support the blood pressure-lowering effects of CGA, showing reduced blood pressure in people taking CGA, with no apparent side effects [47,48]. However, there are conflicting reports that emphasize the need for additional clinical research to fully understand the blood pressure-lowering effects of CGA in humans.

In many other studies, the strong antimicrobial properties of CGA against a wide range of microorganisms, including bacteria, molds, and viruses, have been reported. CGA can effectively inhibit the growth of various bacterial pathogens (e.g., Streptococcus pneumoniae, Staphylococcus aureus, Bacillus subtilis, Escherichia coli, Shigella dysenteriae, and Salmonella Typhimurium) with a minimum inhibitory concentration (MIC) of 20 to 80 µg/mL [49]. The antimicrobial effects of CGA extend to Gram-positive and Gram-negative bacteria and affect cell cycle progression, metabolism, and normal cellular activities by increasing membrane permeability, disrupting cell membrane structure, and leaking intracellular components, which ultimately lead to irreversible changes in membrane potential and cell death [25]. Studies also emphasize the antifungal activity of CGA against Candida spp. [50] and its antiviral effects against herpes simplex virus (HSV) [51], human immunodeficiency virus (HIV) [52], adenovirus [53], and Ebola virus [54]. While CGA shows strong antimicrobial activities, further research is needed to investigate the stability, potential adverse effects, and specific roles of CGA and its metabolites in health promotion.

In addition to the above-mentioned promising health-promoting benefits of CGAs, several studies have been reported their potential cognitive and neuroprotective effects [55,56] and prebiotic properties [11,12]. However, it should be noted that only about one-third of CGAs are absorbed into the human bloodstream, and the rest reach the large intestine and undergo extensive metabolism by colonic microbiota, resulting in other compounds, such as ferulic acid, caffeic acid, and so on [57]. Thus, further studies are necessary to determine whether the health benefits associated with CGA are directly from its compositions or metabolites.

## 3. The Mechanism of Protein–CGA Interactions

CGAs may interact with proteins by either covalent linkages or non-covalent interactions, each influencing the techno-functional and biological properties of the resulting conjugates or complexes, respectively. The covalent interactions between proteins and CGAs involve chemical bond formation that usually occurs via C–S or C–N linkages, which are characterized by a high degree of stability and irreversibility. Conversely, non-covalent interactions, such as hydrogen bonding, hydrophobic attraction, electrostatic interactions, and van der Waals forces, are typically weaker and reversible [15]. Covalent and non-covalent interactions between proteins and phenylic compounds can be characterized using various approaches, such as turbidity measurement, dynamic light scattering, fluorescence intensity distribution, Folin–Ciocalteu, NMR and Fourier transform infrared (FTIR) spectroscopy, reversed-phase high-performance liquid chromatography (RP-HPLC), and sodium dodecyl-sulfate polyacrylamide gel electrophoresis (SDS-PAGE) methods, as well as molecular docking and molecular dynamic (MD) simulations. Understanding the mechanism of these interactions can help to optimize the production and utilization of protein–CGA conjugates and complexes in various food formulations and biomedical applications.

### 3.1. Covalent Interactions (Conjugates)

In principle, covalent interaction between proteins and phenolic acids can be achieved using various chemical approaches, including alkaline, free radical, and enzymatic methods. However, only a few of these methods are widely used in protein–CGA conjugates and suitable for large-scale production.

#### 3.1.1. Alkaline Method

**Figure 4:**
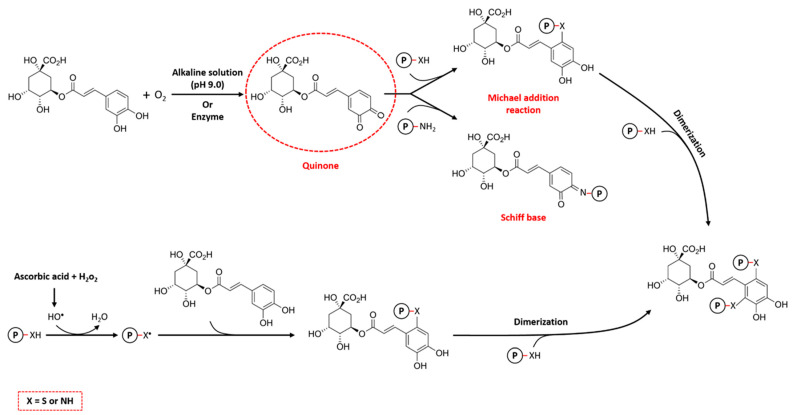
The mechanisms of covalent reactions between proteins and chlorogenic acids (CGAs).

The alkaline method is the most widely used non-enzymatic technique for the formation of protein–CGA conjugates due to its simplicity, mild reaction conditions, and cost-effectiveness. The basic principle of the alkaline method involves the oxidation of polyphenols to quinones under alkaline conditions (pH 9.0). In this process, CGAs are easily oxidized in the presence of oxygen to form semi-quinone radicals, which subsequently rearrange to quinones. After that, nucleophilic residues in protein, such as cysteine, histidine, lysine, tryptophan, methionine, and tyrosine, bind with these reactive intermediate products via Michael addition and/or Schiff base mechanisms (Figure 4). This binding leads to the formation of covalent cross-links, mainly through C–S or C–N bonds, between the CGAs and amino acid residues on the proteins [2,58]. In addition, during the Michael addition reaction, CGAs may undergo a second oxidation, leading to the re-formation of the quinone and potential reaction with a nucleophilic group on another protein, leading to cross-linking of the protein, which is called dimerization [59].

In general, the turbidity and molecular weight (MW) of the protein–CGA conjugates increased as the molar ratio of CGA to protein increased in the system, which may be due to the formation of micro-aggregations with higher particle sizes [60,61]. Furthermore, the interactions between proteins and phenolic acids can be determined by changes in fluorescence intensity, which is an indicator of tryptophan content. In this regard, Elsebaie and Ali [62] showed that the tryptophan content of whey protein isolate (WPI) was reduced up to 58.72 and 85.03% when it covalently interacted with CGA and rosmarinic acid (RC), respectively. Almost similar findings were reported by Lin et al. [63], who showed lower fluorescence intensity for 7S-epigallocatechin-3-gallate (EGCG) and -CGA conjugates using the alkaline method. These results indicate the aggregation of proteins after conjugation with phenolic compounds, which may cover the hydrophobic amino acids and thus reduce the fluorescence intensity. On the other hand, the RP-HPLC method is another widely used technique to semi-quantify protein–CGA conjugates. For instance, Jia et al. [64] evaluated the covalent interactions between sunflower protein isolate (SFPI) and CGA according to their absorbance at 330 nm after 28 (i.e., peak I) and 29 (i.e., peak II) minutes. The areas of both peaks became larger above an SFPI-CGA ratio of 1:1. Moreover, a plateau was obtained approximately at a molar ratio of 1:10, which indicates that the majority of SFPIs (or available binding sites) were conjugated with the CGAs. Overall, these quantitative and non-quantitative methods can be used to detect the optimal ratio of proteins and CGAs in food systems.

#### 3.1.2. Free Radical Method

In the free radical method, which is also referred to as the free radical-induced grafting method, ascorbic acid (C_6_H_8_O_6_) and hydrogen peroxide (H_2_O_2_) are utilized as a redox pair to functionalize proteins, followed by reaction with CGAs to form the conjugate. As illustrated in Figure 4, ascorbic acid is initially oxidized by H_2_O_2_ to form hydroxyl radicals, which can oxidize the amino groups of proteins and produce protein radicals. These macroradicals then covalently interact with CGAs, resulting in the formation of protein–CGA conjugates. Similar to the alkaline method, these obtained conjugates also have the potential to undergo secondary addition reactions to form cross-linked protein polymers [15,65]. In this regard, Fan et al. [66] prepared the conjugates of β-lactoglobulin (β-LG) and CGA using the free radical method to initiate the reaction. They demonstrated that as the concentration of CGA increased from 0.01 to 1, the conjugation amounts enhanced from 31.3 mg g^−1^ to 107.7 mg g^−1^, leading to the higher antioxidant activity of protein–CGA conjugates. However, it should be noted that the relevant conjugation efficiency (i.e., CGA content in conjugates/total amount of CGA, g g^−1^) decreased from 32.4 to 12.1% with the increase in the CGA ratio in the system. On the other hand, a relatively lower conjugation efficiency (8.8%) was reported by He et al. [67] for those wheat gluten hydrolysate (WGH)–CGA conjugates produced by the free radical method, which may be due to the difference in the structure of the proteins. The total phenolic content is another important factor that expresses the ability of proteins to bind CGAs, using the Folin–Ciocalteu method. In this regard, Sun et al. [68] represented that the covalently linked egg white protein (EWP)–CGA conjugates have higher phenolic content (7.13%) compared to the EWP–catechin conjugates (2.76%), using the free radical method. This result indicates that CGA binds to EWPs more easily than catechin due to differences in their molecular weight, methylation, hydrogenation, or hydroxylation. Overall, the structural features of phenolic compounds can strongly affect the protein–polyphenol interactions.

#### 3.1.3. Enzymatic Method

In the enzymatic process, covalent bonds are formed between phenolic compounds, such as CGAs, and proteins with the action of enzymes, such as laccase, tyrosinase, and polyphenol oxidase. This mechanism closely resembles the process observed in the alkaline method (Figure 4). The quinones, generated by enzymatic action, freely interact with the amino acid residues of proteins, resulting in the formation of cross-linked proteins or polymers [58]. In a comparative study, Pan et al. [17] prepared rice protein hydrolysate (RPH)-CGA conjugates using enzymatic, radical-free, and alkaline methods. CGA covalently interacted with the nucleophilic amino acid side chains of RPH, especially the LYS127 site, leading to changes in the structural and functional properties of the protein. Notably, the conjugate formed through enzymatic and alkaline methods exhibited superior antioxidant capacity than those formed through the radical-free approach, indicating the antioxidant potential and health benefits of enzymatically synthesized conjugates in food and non-food sectors. However, high cost, complexity, and environmental considerations restrict its widespread commercial applications compared to conventional non-enzymatic methods.

### 3.2. Non-Covalent Interactions (Complexes)

**Figure 5:**
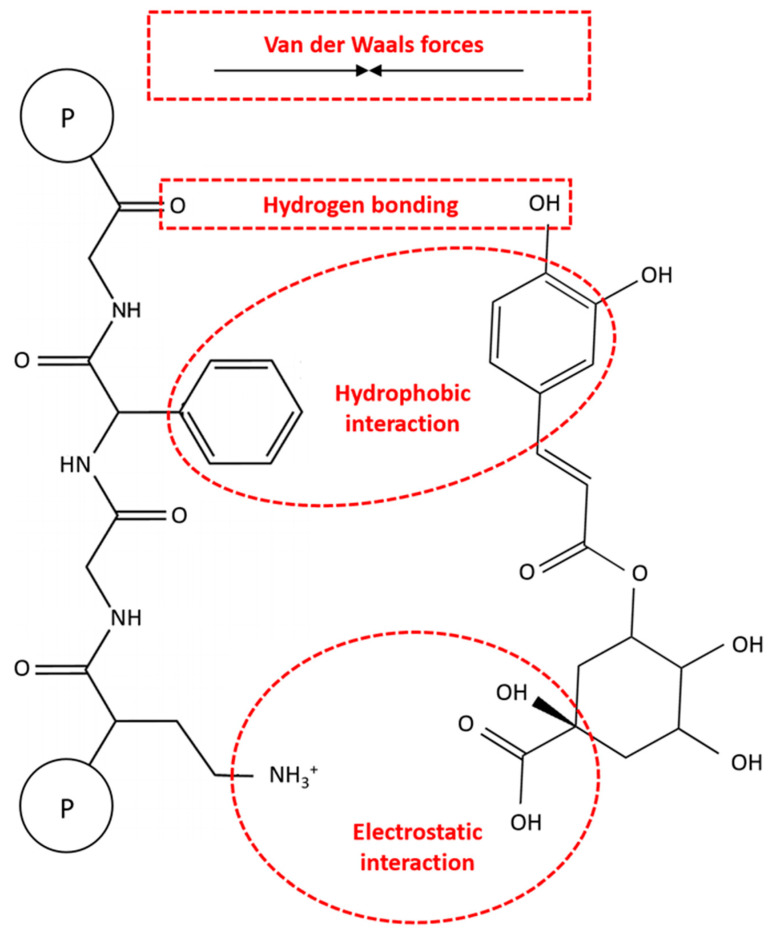
The mechanisms of non-covalent reactions between proteins and chlorogenic acids (CGAs).

Non-covalent interactions involve weak intermolecular forces like hydrogen bonding, hydrophobic interactions, van der Waals forces, and electrostatic interactions. These interactions are reversible and lack the formation of chemical bonds, primarily occurring between the amino groups of proteins and the hydroxyl groups of CGAs (Figure 5). These reversible interactions are more studied than their irreversible ones due to the challenges in quantitating covalent bonds between molecules [16].

Like other protein–polyphenol complexes, hydrogen bonding and hydrophobic interactions are the dominant non-covalent interactions between proteins and CGAs [69,70]. Hydrogen bonding involves the bonding between the hydrogen-donating capabilities of CGAs and the carboxyl groups of proteins. In this respect, the polar amino acid side chains of proteins, such as serine, tyrosine, threonine, and asparagine, bond with the hydroxyl groups of CGAs. On the other hand, the hydrophobic interactions can be caused by the binding of hydrophobic or non-polar amino acids (e.g., alanine, valine, leucine, proline, tryptophan, glycine, methionine, isoleucine, and phenylalanine) to the non-polar aromatic rings of CGAs [14,15]. In this regard, Ren et al. [71] reported that three hydrogen bonds are formed between the hydroxyl groups of CGA and GLN59, VAL43, and TRP19 of β-LG with bond distances of 2.18, 2.68, and 2.71 Å, respectively. These results were also confirmed with the FTIR spectra analysis, indicating that hydrogen bonds were formed between CGA and β-LG. In another study, Zhou et al. [72] revealed that under an acidic condition (i.e., pH 6.0), CGA binds to eight amino acid residues of myofibrillar protein (MP), including ASN50, ASN156, ASP157, ASN158, SER160, THR109, LYS108, and LYS596 through hydrogen bonding. On the other hand, under an alkaline condition (i.e., pH 8.5), CGA exhibited strong interactions with other binding sites of MP, such as ASN50, LEU55, TYR58, ASN156, Gly105, TYR114, and PRO51. The non-covalent interactions between MP and CGA under alkaline conditions can also be involved in hydrophobic forces. Therefore, these findings show that not only the type of protein but also the environmental conditions can play a crucial role in protein–CGA complexes.

Van der Waals forces are the other important non-covalent interactions in protein–CGA complexes, which are affected by the ambient solvent conditions. These forces primarily include dipole–induced dipole and induced dipole–induced dipole interactions between neutral atoms in protein molecules. The resulting interactions between these induced dipoles can include both attractive and repulsive components, depending on the pH of the environment, as well as the relative number of positively and negatively charged residues. Moreover, electrostatic interactions may occur between positively charged protein groups and negatively charged CGA groups, although these are less common due to the typically uncharged state of CGAs under normal food pH conditions [16]. In a comparative study by Liu et al. [73], the binding behaviors of CGA with five milk proteins, namely α-, β-, and κ-casein, as well as α-lactalbumin (α-LB) and β-LG, were indicated. Thermodynamic parameters showed that hydrogen bonding and van der Waals forces are the main interactions in CGA-α-casein and CGA-α-LB complexes, while hydrophobic interactions were dominant in other complexes. Moreover, Zhou et al. [74] showed that hydrogen bonds and van der Waals forces are the major driving forces promoting the binding of CGA and EGCG to soybean 7S globulin. These complexes could also be affected by electrostatic interactions, depending on the structure and molecular weight of polyphenols. On the other hand, Wang et al. [75] demonstrated that hydrogen bonding and electrostatic interactions are the primary non-covalent interactions between zein and CGA based on the FTIR spectra analysis. Overall, the actual mechanism of non-covalent reactions of CGA with proteins from different sources is still unknown, and further research is needed for their effective food and non-food applications.

## 4. Changes in Protein Characteristics

### 4.1. Animal Protein–CGA Interactions

The covalent and/or non-covalent interactions between animal proteins and CGA can cause some changes in the secondary and tertiary structures of proteins, which ultimately affect their techno-functional and health-promoting attributes, as shown in Table 1. These changes are highly dependent on the type, size, and conformation of the protein, the pH and temperature of the environment, and the type of the involved interactions. Moreover, the characteristics of conjugates and complexes, whether bound covalently or non-covalently, exhibit a dose-dependent relationship with CGA. Therefore, it is important to understand how these factors impact the final functionality of animal protein–CGA complexes or conjugates under different conditions.

#### 4.1.1. Milk Proteins

Polyphenol-rich food products, such as coffee, chocolate, and tea, are usually consumed with milk. Some previous studies have focused on the dietary absorption and antioxidant activity of polyphenols when consumed with milk. Generally, the interactions between proteins and polyphenols are considered as the main reason for the effect of milk proteins on the absorption and antioxidant activity of polyphenols [73,76]. However, little is known about the influence of individual polyphenols, such as CGA, on the structural, physicochemical, functional, and biological properties of each major milk protein, namely whey proteins and caseins, which will be discussed in this section.

##### Whey Proteins

Whey proteins are a series of globular proteins, mainly α-LA and β-LG, which have received much attention in the food industry due to their excellent encapsulating, structuring, emulsifying, foaming, and gelling properties. However, their industrial applications are limited due to the dependence of their performance on mineral levels, pH, and temperature [2]. Recent studies have demonstrated the potential application of CGA to improve the techno-functional properties of WPI and its fractions (i.e., α-LA and β-LG), as well as their biological attributes. For instance, Meng and Li [77] evaluated the structural and functional properties of WPI before and after binding with CGA, EGCG, and gallic acid (GA). The results showed that the presence of these polyphenols enhanced the solubility, foaming, and emulsifying capacities of WPI. Specifically, the interactions between WPI and CGA led to significant structural changes in the complexes, characterized by a decrease in β-sheet and α-helix and an increase in β-turn and random coil contents in FTIR spectroscopy, as well as a red-shifted maximum emission wavelength in intrinsic fluorescence spectroscopy. In another study, Elsebaie and Ali [62] reported that the covalent interactions between WPI and CGA or RA resulted in significant changes in the structural properties of the protein. Additionally, the conjugation of WPI and these polyphenols significantly increased the antioxidant, antimicrobial, and anticancer activities of the protein, suggesting the potential health-promoting effects of the modified proteins. In a further study, Xu et al. [78] found that the covalent conjugation of WPI with CGA unfolded the structure of the protein, which resulted in WPI-CGA conjugates with lower IgE binding activity but higher intestinal digestibility than unmodified WPI, suggesting a promising approach to reduce the allergenicity of WPI. Furthermore, the solubility, emulsifying activity, foaming properties, and antioxidant capacity of WPI were enhanced by the addition of CGA. These findings indicate the potential application of CGA to modify the functional and biological properties of WPI while reducing its allergenicity.

In a comparative study, Zhang et al. [70] investigated the non-covalent binding interactions between the two fractions of WPI (i.e., α-LA and β-LG) and CGA using spectroscopic and molecular docking analysis. The authors found that CGA quenched the fluorescence of the two whey proteins through a static mode, with the binding affinity declining in the order of α-LA > β-LG. The results also revealed that hydrophobic interactions and hydrogen bonds contributed to the major interactions between the proteins and CGA. Additionally, the interactions with CGA led to the unfolding of α-LA and β-LG and reduced their surface hydrophobicity, which ultimately affects the functional properties of the proteins. In another study, Qie et al. [79] explored the interactions between β-LG and CGA under different thermal treatments (25–121 °C). At temperatures below 60 °C, non-covalent interactions, e.g., hydrogen bonding and hydrophobic forces, formed between CGA and β-LG. However, as the temperature increased from 85 to 121 °C, covalent bonds were the main interactions between β-LG and CGA due to changes in the secondary structure and unfolding of the protein. The results also revealed that the thermal stability of β-LG was enhanced through both covalent and non-covalent interactions with CGA. On the other hand, the interactions between β-LG and CGA significantly increased the antioxidant activity of CGA by 56.18 and 23.26% according to the 2,2′-azino-bis(3-ethylbenzothiazoline-6-sulfonic acid) (ABTS) and ferric reducing power (FRAP) assays, respectively, compared to untreated CGA. These findings provide insights into the structural, physicochemical, and antioxidant properties of β-LG-CGA complexes/conjugates, which are important for their further applications as functional ingredients in various food sectors, especially the dairy industry.

##### Casein Proteins

Casein proteins, the main phosphoproteins of mammalian milk, exist as micelles of αS1-, αS2-, β-, and κ-caseins. β-casein (β-CN) is the most abundant type of casein (~35% of bovine casein) with a molecular weight of 24.0 kDa, which is widely utilized in ice cream, cheese, and meat products. β-CN is also considered as a suitable carrier for the delivery of polyphenols due to the presence of both hydrophilic and hydrophobic binding sites in its structure [80,81]. In a study by Yin et al. [82], the physicochemical and antioxidant properties of β-CN-CGA complexes were investigated during the thermal treatments at 25–121 °C. This study showed that CGA mainly binds to β-CN through hydrophobic interactions, and thermal treatment at 121 °C weakened the binding affinity between these two compounds. Moreover, the complexes formed between β-CN and CGA after thermal treatments did not show any covalent bonds. On the other hand, after 30 min of thermal treatment at 65 °C, β-CN-CGA complexes exhibited rather strong antioxidant activity in both FRAP and ABTS assays. Furthermore, Jiang et al. [83] found that the solubility, foaming, digestibility, and radical scavenging activity of casein were improved by the addition of CGA, especially at medium and high concentrations (i.e., 120–240 μM/g protein). These results support the potential use of CGA in functional dairy beverages considering its techno-functional and health benefits.

#### 4.1.2. Myofibrillar Proteins

Myofibrillar proteins (MPs) are mainly responsible for the texture, appearance, and organoleptic properties of meat products. During processing and storage, protein oxidation often leads to unpleasant changes in the physicochemical and functional characteristics of meat products, which can be controlled by various modification techniques, such as the complexation/conjugation of MPs with polyphenols. [84]. For instance, Guo et al. [85] investigated the effects of CGA on the techno-functional properties of Coregonus peled MP through hydroxyl radical oxidation. The results revealed that CGA can significantly inhibit the increase in protein carbonyl content but did not prevent free amine and sulfhydryl losses caused by oxidation. Moreover, oxidized MPs containing 6 µM/g CGA exhibited better functional properties, while those containing 150 µM/g CGA formed aggregates, resulting in insolubility and poor gel networks. These results are well aligned with the previous study by Cao and Xiong [86]. They also found that the addition of CGA intensified the oxidation-initiated loss of the α-helix conformation as well as the tertiary structure of MP. In another study, Chang et al. [87] evaluated the influences of CGA and transglutaminase (TGase) on the physicochemical and gel properties of pork MP. The results showed that TGase improved the gel structure of CGA-oxidized MPs, especially at lower CGA concentrations. The addition of TGase regularized the gel network of the protein and overcame its excessive aggregation caused by high doses of CGA. Furthermore, this study demonstrated that TGase dramatically enhanced the water-holding capacity (WHC) of oxidized MPs treated with CGA, leading to uniform moisture distribution. Overall, this study highlighted that the addition of TGase positively contributes to the textural properties and WHC of protein gels under oxidative conditions and overcomes the technical limitations of using CGA in MPs at high concentrations.

The interactions between protein and CGA not only improve the techno-functional and biological properties of proteins but also can affect the health-promoting effects of CGAs. In this respect, Zhou et al. [72] showed that the antioxidant activity and bio-accessibility of CGA were significantly enhanced after the complexation with MP. Additionally, the authors found that the MP-CGA complexes formed at pH 6.0 showed significantly higher concentrations of piplartine and rhetsinine, two well-known compounds to modulate diabetes, compared to those formed at pH 8.5. In a further study, Zhou et al. [88] found that the MP-CGA complexes can improve hyperlipidemia and hyperglycemia and reduce intestinal inflammation and intestinal barrier injury in type 2 diabetic mice. This study showed that MP-CGA reconstructed the gut microbiota in these mice, increasing the abundance of probiotics Parabacteroides, Akkermansia, and Bacteroides, while suppressing the opportunistic pathogens Staphylococcus and Enterococcus. Additionally, MP-CGA complexes significantly increased the concentration of intestinal metabolites, e.g., butyric acid, which positively regulates type 2 diabetes mellitus (T2DM). The results also highlighted that the effect of MP-CGA complexes on blood glucose regulation was more pronounced than that of CGA alone, suggesting new opportunities for the application of polyphenols combined with meat proteins in the treatment of T2DM.

Overall, these studies highlight the importance of protein–CGA interactions in meat products, indicating that the addition of CGA can delay/prevent oxidation degradation, improve emulsifying and foaming capacity, and enhance the gelling properties of MPs. On the other hand, the results show the potential of MP-CGA complexes to enhance the bio-functional properties of proteins and polyphenols, particularly in acidic environments.

#### 4.1.3. Human and Bovine Serum Albumins

Serum albumin, commonly recognized as a major plasma protein (50–60%), is ubiquitously distributed among vertebrates, indicating its widespread accessibility and versatility. Human and bovine serum albumins have been widely used in tissue engineering and regenerative medicine since the mid-1990s [89]. On the other hand, these proteins can bind with a variety of substrates, including polyphenols, fatty acids, amino acids, and metal cations. Furthermore, it has been demonstrated that the concentration, distribution, and metabolism of various drugs can be significantly altered as a result of their binding to serum albumin [90,91]. Therefore, investigating the interactions of CGA with human and bovine serum albumins can provide useful information about their actions and pharmacokinetic properties. In this respect, Hu et al. [92] investigated the specific binding sites of CGA to human serum albumin (HSA) using fluorescence and UV-Vis absorption spectroscopy. The results revealed that CGA forms a complex with HSA, binding specifically to site I (subdomain IIA), which is also known as the Sudlow I binding site. Moreover, the interaction between CGA and HSA could affect the thermal stability of the protein. Similarly, Tang et al. [93] revealed that CGA and its two isomers, namely cryptochlorogenic acid (CCGA) and neochlorogenic acid (NCGA), bind to HSA at the Sudlow I site and affect the secondary structure of the protein. On the other hand, the authors reported that CCGA had the strongest ability of form hydrogen bonds, while CGA and NCGA generated more electrostatic interactions with HSA. These findings contribute to the understanding of the structure–affinity relationship between CGA and HSA, which can be useful in the design of novel polyphenol-like drugs and phenolic food additives.

In addition to HSA, the interaction between bovine serum albumin (BSA) and CGA has also been investigated in previous research. For instance, Rawel et al. [94] reported structural changes in BSA due to the covalent interactions with CGA, leading to a decrease in α-helix and an increase in other structures, e.g., β-strand and β-turn. The interactions between BSA and CGC also resulted in a shift in the isoelectric point of BSA to lower pH values. Moreover, the hydrophobic–hydrophilic balance of BSA was modified, which can improve its techno-functional properties, such as solubility, emulsion, and foam properties. Moreover, Zhang et al. [95] showed that the binding affinity of CGA with BSA increased by 11 and 27 times in the presence of Cu^2+^ and Al^3+^, respectively, which may provide some promising information for the clinical application of drugs in the pharmacy, pharmacology, and biochemistry industries. However, further in vivo studies are required to investigate the actual ternary interactions between polyphenols, metal ions, and proteins in the human body.

#### 4.1.4. Egg White Proteins

Egg white is mainly composed of water (88%) and protein (11%), with the rest being carbohydrates, lipids, and ash. On the other hand, ovalbumin (54%), ovotransferrin (12%), ovomucoid (11%), ovomucin (3.5%), and lysozyme (3.5%) are considered as the main proteins found in egg white [96]. Egg white proteins (EWPs) are widely used as an emulsifying agent in the food industry due to their excellent surface activity and emulsion stabilization properties. However, these proteins generally exhibit poor oxidative stability, which limits their further applications. In this regard, some studies have reported the promising potential of polyphenols, especially CGA, to improve these shortages of EWPs. For instance, Sun et al. [68] found that the incorporation of CGA into EWP resulted in improved antioxidant activities, with a 4.9-fold higher FRAP and 4.5-fold higher 2, 2-diphenyl-1-picrylhydrazyl (DPPH) free radical scavenging activity compared to unmodified EWP. The interaction between EWP and CGA was primarily through cysteine, tyrosine, and tryptophan residues, and the conjugation shifted the isoelectric point of EWP to the lower pH values. In addition, the results indicated better emulsification properties for the EWP-CGA conjugates, which was attributed to a decrease in surface hydrophobicity. In another study, Sun et al. [97] prepared dual-function emulsifiers with improved antioxidant and emulsifying properties by the conjugation of five polyphenols, e.g., CGA, EGCG, caffeic acid, catechin, and quercetin to EWP using an ultrasound-assisted free radical method. This study revealed that ultrasound treatment can accelerate the covalent interactions between EWP and CGA and other polyphenols by enhancing the production of hydroxyl free radicals and the dispersion of EWP and polyphenols. These interactions significantly improved the antioxidant and emulsifying properties of EWP, especially when modified by CGA with a C3-C6 structure. Moreover, Ren et al. [71] demonstrated that the antioxidant activity of ovalbumin (OVA) increased with the addition of CGA. Moreover, the OVA-CGA conjugates showed a lower allergenic capacity, indicating the covalent conjugation of proteins and polyphenols, such as CGA, as a promising way to reduce the allergenicity of proteins. In a further study, Perumal et al. [98] explored the non-covalent binding mechanisms between CGA and OVA through multi-spectroscopic studies and in silico simulations. The authors found that CGA forms hydrogen bond interactions with specific residues of OVA, e.g., L101, S100, F99, S98, N94, K92, T91, and N88 residues, leading to a decrease in the α-helix content of the OVA structure. This study also revealed that the OVA- CGA complexes are formed through both hydrogen bonding and electrostatic interactions, which can significantly affect the bioactivities, metabolism, and absorption of CGA. These findings provide useful fundamental information on the application of the OVA-CGA complex in the food and pharmaceutical industries.

#### 4.1.5. Others

In addition, the interactions of porcine plasma protein (PPP) and gelatin with CGAs have also been investigated in some studies. For instance, Chen et al. [99] found that the addition of oxidized chlorogenic acid (OCA) could significantly improve the physical stability of stabilized porcine plasma protein hydrolysates (PPPHs) emulsions, by inducing higher absolute ζ-potentials, lower coalescence indices and flocculation factors, and smaller particle sizes, as well as producing thicker interfacial films around each oil droplet. Moreover, OCA inhibited both lipid and protein oxidation in a concentration-dependent manner, indicating better oxidative stability and antioxidant activity of the PPPH-stabilized emulsions. In a further study by Chen et al. [100], they evaluated the techno-functional and antioxidant properties of PPPHs modified with OCA in comparison with those modified with oxidized tannic acid (OTA). This study showed that both OCA and OTA were covalently attached to PPPHs, leading to increased emulsifying stability and greater antioxidant activities. However, the emulsifying activity indices of PPPHs significantly decreased after the addition of OCA and OTA, which was associated with their lower surface hydrophobicity values. Moreover, the interactions between PPPHs and OCA resulted in lower turbidity compared to PPPH-OTA conjugates, indicating the greater ability of OCA to bind to PPPH molecules. Therefore, these results indicate that PPPH-OCA conjugates can be used as efficient antioxidants and potential emulsifiers in emulsion-based food systems. In another study, Fu et al. [101] also investigated the interactions between CGA and gelatin at the optimal reaction molar ratio of 6:1, 4:1, 2:1, 1:1, 1:2, and 1:4. According to the results, when the reaction ratio of CGA to gelatin reached 4:1, the grafting ratio and CGA content in the conjugates was 94.20 ± 0.68% and 84.78 ± 0.61 mg CGA g-1 gelatin, respectively, which was higher than the other ratios, indicating the optimal reaction molar ratio for the production of CGA–gelatin complexes. Furthermore, the resulting CGA–gelatin complexes exhibited higher antioxidant activity than free CGA and showed antibacterial activity against both Gram-positive and Gram-negative bacteria, indicating their potential to be used as novel coating and film materials.

**Table 1:** Summary of studies on animal protein–CGA interactions.

Protein Type	Binding Type	Preparation Method	CGA Concentration	Main Results	References
WPI	Non-covalent	Mixing in phosphate buffer	500 μM	□ Enhanced solubility, foaming, and emulsifying properties □ Lower surface hydrophobicity	[77]
	Covalent	Alkaline method	NS	□ Enhanced antioxidant, antimicrobial, and anticancer activities □ Lower tryptophan content	[62]
	Covalent	Free radical method	0.5 g	□ Reduced IgE binding activity and allergenicity □ Enhanced solubility, foaming, emulsifying, and antioxidant properties	[78]
α-LA and β-LG	Non-covalent (i.e., H-bond and hydrophobic)	NS	2, 4, 8, 16, 32, and 64 µM	□ Decreased the binding affinity in the order of α-LA > β-LG □ Lower surface hydrophobicity	[70]
β-LG	Covalent and non-covalent	Mixing in phosphate buffer	1.5 mM/150 μM Pr	□ Enhanced thermal stability and antioxidant activity	[79]
CN	Non-covalent	Mixing in phosphate buffer	20, 120, and 240 µM/g Pr	□ Enhanced solubility, foaming, digestibility, and antioxidant activity □ Lower surface hydrophobicity	[83]
β-CN	Non-covalent (i.e., hydrophobic)	Mixing in phosphate buffer	5, 10, 20, 30, 40, and 60 μM	□ Thermal treatment at 121 °C weakened the binding affinity □ High antioxidant activity after 30 min of thermal treatment at 65 °C	[82]
Coregonus peled MP	Covalent and non-covalent	Mixing in Tris-HCl buffer	6, 30, and 150 µM/g Pr	□ Inhibited the increase in Pr carbonyl content □ Altered the structural properties □ Enhanced solubility, foaming, emulsifying, and gelling properties up to CGA concentration of 6 μM/g	[85]
Pork MP	Covalent and non-covalent	Mixing in PIPES buffer	6, 30, and 150 µM/g Pr	□ Inhibited the increase in Pr carbonyl content □ Altered the structural properties □ Enhanced gelling properties up to CGA concentration of 30 μM/g	[86]
	Covalent	Enzymatic method	5 and 20 µM/g Pr	□ Enhanced gelling properties and WHC	[87]
	Non-covalent (i.e., H-bond and hydrophobic)	Mixing in PIPES buffer	150 µM/g Pr	□ Enhanced antioxidant activity and bio-accessibility of CGA	[72]
	Non-covalent	Mixing in PIPES buffer	2.65 mg/10 mg Pr	□ Reduced intestinal inflammation and intestinal barrier damage in type 2 diabetic mice □ Increased the abundance of probiotics and intestinal metabolites, e.g., butyric acid	[88]
HSA	Non-covalent (i.e., H-bond and electrostatic)	NS	NS	□ CGA forms a complex with HSA from site I (subdomain IIA)	[92,93]
BSA	Covalent	Alkaline method	CGA/Pr (*w*/*w*) ratios of 1:2, 1:3, 1:5, 1:7, and 1:10	□ Altered the structural properties □ Reduced denaturation temperature and enthalpy □ Lower surface hydrophobicity	[94]
EWP	Covalent	Free radical method	0.1 g/100 mL Pr	□ Enhanced emulsification properties and antioxidant activity □ Lower surface hydrophobicity	[68]
	Covalent	Free radical method + ultrasound treatment	0.1 g/100 mL Pr	□ Enhanced emulsification properties and antioxidant activity	[97]
OVA	Covalent	Free radical method	0 0.08 g/0.2 g Pr	□ Enhanced antioxidant activity □ Reduced allergenic capacity	[71]
	Non-covalent (i.e., H-bond and electrostatic)	Mixing in phosphate buffer	5 mL, 5 × 10^−4^ M dm^−3^/3 mL, 5 × 10^−6^ M dm^−3^ Pr	□ Affected the bioactivities, metabolism, and absorption of CGA	[98]
PPPH	Covalent	Mixing in phosphate buffer	0.1, 0.5, 1, and 1.5%/40 mg Pr	□ Improved physical and oxidative stabilities □ Enhanced emulsifying stability and antioxidant activity □ Reduced emulsifying activity □ Lower surface hydrophobicity	[99,100]
Gelatin	Non-covalent	Mixing in phosphate buffer	CGA/Pr M ratios of 6:1, 4:1, 2:1, 1:1, 1:2, and 1:4	□ Enhanced antioxidant and antimicrobial activities	[101]

CGA: chlorogenic acid; Pr: protein; PPPH: porcine plasma protein hydrolysate; EWP: egg white protein; OVA: ovalbumin; HSA: human serum albumin; BSA: bovine serum albumin; NS: not stated; MP: myofibrillar protein; PIPES: piperazine-N,N′-bis(2-ethanesulfonic acid); WPI: whey protein isolate; WHC: water-holding capacity; CN: casein; α-LB: α-lactalbumin; β-LG: β-lactoglobulin.

### 4.2. Plant Protein–CGA Interactions

Previous studies indicate that CGAs exhibit diverse effects on plant proteins, such as stabilizing proteins through the formation of complexes/conjugates and protecting them from denaturation due to heat or environmental stressors, as well as reducing the oxidative damage to plant proteins. The interactions between plant proteins and CGAs can also cause structural changes, potentially influencing key functional aspects of proteins, like solubility, emulsification, and gelation behavior. Notably, the impact of CGAs on these techno-functional properties of proteins can vary based on the specific protein and environmental conditions, as well as the CGA concentration. Moreover, CGAs may affect the digestibility and bioavailability of plant proteins by modulating enzymatic hydrolysis and absorption in the gastrointestinal tract through complex formation (Table 2) [25,102]. This section delves into the intricate covalent or non-covalent interactions between CGAs and plant proteins, emphasizing various protein sources.

#### 4.2.1. Soy Protein

Among plant proteins, soy protein isolate (SPI) stands out as the most extensively researched regarding its interactions and the formation of complexes with CGAs due to the presence of high amounts of hydrophobic or non-polar amino acids in its structure, which is an important factor affecting the binding affinity with CGA [103]. In this regard, Budryn et al. [104] showed the highest binding affinity of CGAs with soy protein hydrolysate (SPH), followed by whey protein hydrolysate (WPH) and egg ovalbumin hydrolysate (EOH). These results may be due to the presence of high amounts of arginine, phenylalanine, and proline in SPH, which can increase the hydrogen bonding and hydrophobic interactions in the complex. Additionally, they suggested that the destruction of soy protein throughout the hydrolysis process exposed more hydrophobic groups for interaction with CGA. The higher average peptide length of SPH compared to the other protein hydrolysates can also provide more capacity to form hydrogen bonds between SPH and CGAs. In another study, Guo et al. [105] reported that the formation of covalent conjugations between SPI and CGA improved flexibility and radical-scavenging activity and decreased hydrophobicity in the protein, which was positively correlated with CGA concentration. However, some functional properties, e.g., emulsifying and foaming capacity, as well as solubility, increased only up to a CGA concentration of 80 μmol/g protein and then decreased with higher CGA concentrations. In a further study, Lin et al. [63] reported an enhancement in antioxidant activity, emulsification, foaming capacity, and foam stability of 7S protein after covalent binding with CGA. Moreover, the findings of their in vitro and in vivo analyses revealed a significant reduction in protein allergenicity after covalent modification with CGA. Additionally, in a recent study, Zhou et al. [74] investigated the non-covalent interactions between CGA and soybean 7S globulin, highlighting hydrogen bonds and van der Waals forces as the primary binding mechanisms. In this respect, CGA induced conformational changes in the polypeptide chain of 7S, converting β-sheets to random coils and partially unfolding the protein structure, resulting in reduced surface hydrophobicity. In summary, the outcomes of studies on SPI-CGA interactions vary, depending on the nature of the proteins involved and the driving forces at play. While enhancements in biological and functional properties are often observed, deteriorations in certain traits may occur in some instances.

#### 4.2.2. Zein

Zein is a corn prolamin, accounting for 44–79% of total corn protein, which can be utilized as a biocompatible, non-toxic, and biodegradable substance in various food products [106]. Wang et al. [75] investigated the effect of CGA on the techno-functional and biological properties of zein in CGA-loaded zein-based nanofiber films. Thermodynamic analysis and FTIR graphs revealed the domination of electrostatic interactions and hydrogen bonds in the formation of protein–CGA complexes. The presence of zein and CGA together resulted in decreased thermal stability but enhanced the mechanical properties, antioxidant capacity, and antibacterial effects of the nanofiber films. Moreover, Xu et al. [60] showed that the type of interactions in the zein–CGA formation (i.e., covalent and non-covalent) can significantly change the second structure and morphology of zein. In this respect, the free amino groups and free sulfhydryl groups in zein significantly reduced after conjugating with CGA, which showed a reaction between CGA and SH groups of the protein. The researchers mentioned two potential reasons accounting for the decline in free amino and sulfhydryl groups. Primarily, it is probable that the notably reactive hydroxyl groups present in polyphenols facilitated interaction with proteins through hydrogen bonding. Conversely, alkaline environments could prompt the oxidation of polyphenols by molecular oxygen into corresponding quinones. These resultant quinones represent highly reactive electrophilic entities capable of forming covalent bonds with active protein groups, including amino or sulfhydryl, via Schiff bases or Michael addition reactions. Therefore, the physicochemical and functional attributes of covalently bound zein–CGA conjugates, such as solubility, surface hydrophilicity, thermal stability, and antioxidant activity, surpassed those of the unmodified zein proteins, which is in line with the other studies on covalent conjugation between plant proteins and CGAs.

#### 4.2.3. Wheat Protein

Wheat protein, commonly known as wheat gluten, is a natural component found in wheat grains, comprising two primary proteins known as glutenin and gliadin. In a study by He et al. [67], they investigated the characteristics of wheat gluten hydrolysate (WGH)–CGA conjugates formed through a free radical grafting method. Their findings revealed that the conjugation with CGA significantly enhanced the thermal stability of WGH, elevating its melting point from 170 °C (second peak for WGH) to 191 °C in the conjugates. Furthermore, WGH-CGA conjugates exhibited improved emulsifying and foaming properties, accompanied by higher surface hydrophobicity. In contrast, physical mixtures of WGH and CGA demonstrated lower surface hydrophobicity compared to WGH alone, attributable to differing interaction mechanisms. The non-covalent interaction between WGH and CGA tended to increase the hydrophilicity of the protein, consequently reducing surface hydrophobicity. Conversely, covalent binding with CGA potentially induced the formation of quinone and partial protein conformational changes, leading to the exposure of hydrophobic sites. Moreover, covalent conjugation with CGA enhanced the antioxidant activity and diminished the allergenicity of the hydrolysates. Consistent with these findings, Zhang et al. [107] observed a reduction in gliadin allergenicity following covalent conjugation with CGA, along with improved thermal stability, in vitro digestibility, and antioxidant activity of gliadin–CGA conjugates. In summary, the literature on wheat protein–CGA interactions indicates enhanced functional properties in the proteins post-covalent binding with the polyphenol.

#### 4.2.4. Rice Protein

Rice proteins are a good option to be used in food products, as they barely show any allergenicity. Rice bran protein (RBP), which is derived from agricultural by-products, has also gained popularity in recent years, due to its low cost, high nutritional value, and sustainability [108,109]. For instance, Wang et al. [110] investigated the non-covalent complex formation between ultrasonically treated rice bran protein (URBP) and CGA. The addition of CGA to create a complex resulted in a red-shift of the complex’s λmax compared to its pre-complexation state. The authors suggested that this red-shift phenomenon likely arose from the formation of intermolecular hydrogen bonds between the hydroxyl groups of CGA and either the sulfhydryl or hydroxyl groups of the protein. The addition of CGA resulted in a decrease in the hydrophobicity index (H0) of the complex compared to untreated RBP. Upon CGA addition, the complex exhibited a decrease in α-helix content from 22.62% to 21.81% and a decrease in β-sheet content from 41.01% to 39.43%. Conversely, there was a significant increase in β-turn content (*p* < 0.05), rising from 17.39% to 18.45%, along with an increase in random coil content from 18.98% to 20.31%. These alterations are attributed to the formation of hydrogen bonds and hydrophobic interactions between CGA and RBP, resulting in the reorganization of the peptide chain and subsequent modification of the RBP’s secondary structure. Their results also showed an increase in the emulsifying activity index (EAI) and emulsion stability index (ESI) of the RBP-CGA complex compared to RBP alone. In another study, Pan et al. [61] explored the covalent interaction between rice protein hydrolysates (RPHs) and CGA under alkaline conditions (pH 9.0). This interaction reduced fluorescence intensity and caused a red-shift. Incorporating 0.025% CGA notably enhanced the emulsifying activity of RPH. The resulting emulsions exhibited enhanced physical stability, with minimal changes in size (0.08 μm) and ζ-potential (3.34 mV) (*p* > 0.05). Additionally, the covalent interaction improved oxidative stability, effectively preventing lipid oxidation during storage. Adequate CGA addition increased hydrolysate adsorption to the emulsion interface, forming a thicker interfacial film around oil droplets. In a further study on RPH-CGA conjugates, it was demonstrated that covalent conjugation led to a rise in the quantity of bound CGA, increasing from 15.23 to 21.11 nmol/mg. Moreover, the levels of random coils, emulsifying activity, and antioxidant capacity of RPH exhibited augmentation following covalent binding with CGA [17]. Collectively, the conjugation with CGA exhibited promising enhancements across multiple properties of rice proteins and their hydrolysates.

#### 4.2.5. Sunflower Protein

Sunflower seeds have a high protein content, ranging from 30 to 50%, and their protein shows considerable techno-functional properties. In fact, both sunflower protein (SFP) and CGA exist in the seed cells and both show high reactivity towards each other. Therefore, in the processing condition of the sunflower seeds for protein/oil extraction, their reaction seems unavoidable [111]. Karefyllakis et al. [112] explored the characteristics of SPF-CGA conjugates formed through covalent bonds. The degree of complexation showed a direct correlation with the concentration of CGA, leading to the formation of cross-links and significantly affecting the secondary and tertiary structure of the proteins. The conformational changes in SFP altered its hydrophobicity by exposing more hydrophobic fractions. However, the highest hydrophobicity was observed at the lowest CGA content, and then it went down continuously as the ratio of CGA increased. The authors noted that this result could indicate that CGA binds more extensively to the protein, possibly due to the polar characteristics of this phenolic compound, which featured an abundance of hydroxyl groups at interfacial binding sites. This additional conjugation resulted in a partial restoration of hydrophilicity levels. Moreover, Jia et al. [64] explored CGA-SFP complexes across molar ratios ranging from 10:1 to 1:10. These complexes formed through both covalent and non-covalent interactions by adjusting the pH levels to 7 or 9. Their findings revealed an improvement in protein solubility regardless of the binding mode. Notably, covalently bonded complexes at molar ratios of 1:1 and higher displayed a change in color. While CGA did not impede gel formation, variations in ratios and binding forces led to differences in gel strengths. The highest gel strength was observed in covalent complexes with a molar ratio of 1:1. In summary, the researchers suggested that removing all CGA fractions from SFP extract may not be necessary, as it could enhance certain protein attributes depending on the intended application. Nevertheless, adjusting CGA concentration could achieve more favorable ratios.

#### 4.2.6. Others

In addition to the above-mentioned proteins, the interactions between CGAs with other plant proteins, such as hemp, quinoa, flaxseed, and pea, have been also investigated. For instance, Yu et al. [113] used a combination of ultrasound treatment and complexation to form hemp protein (HP)–CGA complexes and evaluated their structural and functional properties. This strategy resulted in more significant changes in the structure of polypeptide chains compared to the application of each treatment individually, which ultimately led to the improvement of techno-functional properties of the complexes. In another study, Ji et al. [114] investigated the non-covalent interactions between quinoa protein hydrolysate (QPH) and CGA, by measuring the changes in sulfhydryl content and SDS-PAGE patterns. Their results showed that CGA causes partial unfolding of the quinoa protein structure and reduces its surface hydrophobicity. In addition, this led to an enhanced oxidation ability, resulting in improved antioxidant effects in QPH treated with CGA. Furthermore, interface and emulsification experiments revealed that CGA reinforces the oil–water interfacial activity of QPH, thereby increasing its emulsion stability. In a further study, Cao et al. [115] studied the interactions of flaxseed protein (FP) and CGA in alkali-fabricated conjugates. Their results showed improved antioxidant activities in the conjugates compared to the protein alone. The researchers proposed that covalent bonds formed between CGA and FP led to subtle microstructural alterations in the protein. Additionally, increasing the levels of CGA from 0.35 to 1.40% resulted in higher WHC values, ranging from 409.05 to 500.81%, surpassing that of FP alone (353.03%). Conversely, CGA notably diminished the emulsifying activity index (EAI), surface hydrophobicity, and oil-holding capacity (OHC) of FP. Across CGA concentrations of 0.35% to 1.40%, OHC values ranged from 428.525% to 273.495%, all lower than those of FP alone. On the other hand, FP with 0.70% CA showed the lowest EAI (39.70 cm^2^ g^−1^), indicating a 61.52% reduction in the EAI compared to untreated FP. In a recent investigation, Hao et al. [116] examined the structural and functional attributes of pea protein isolate (PPI) under the influence of non-covalent interactions with CGA. The polyphenol formed stable complexes with PPI and statically quenched the emitted fluorescence of the protein. Notably, the maximum emission peak shifted from 337.6 nm in native PPI to 347.6 nm in the complexed state. The principal driving force behind the interaction between PPI and CGA was electrostatic attraction, resulting in a decrease in free sulfhydryl groups upon the complexation. Moreover, the surface hydrophobicity decreased from 446.27 to 373.57. Furthermore, the complex formation led to enhancements in emulsifying activity, foaming property, and in vitro digestibility of PPI.

It is noteworthy that in some cases, the interaction of a plant protein and CGA is not favored, as the phenolic acid tends to interact with specific amino acids, like methionine and lysine, resulting in lower nutritional value of the final product [117]. However, to date, the findings from existing studies exhibit encouraging improvements in the functional and biological attributes of plant proteins subsequent to their interaction and conjugate/complex formation with CGAs. Moreover, previous studies examining both covalent and non-covalent bindings of proteins with CGAs have revealed that covalent conjugation exerts a more pronounced influence on the structure and techno-functional properties of the proteins. Nonetheless, the existing dataset within this domain remains limited. Therefore, expanding the investigation to encompass additional plant protein sources, including legumes beyond soybean, nuts, and green leaf proteins, could provide a more comprehensive understanding of these interactions.

**Table 2:** Summary of studies on plant protein–CGA interactions.

Protein Type	Binding Type	Preparation Method	CGA Concentration	Main Results	References
SPH	Non-covalent (i.e., H-bond and hydrophobic)	Mixing in phosphate buffer	0.015 g/0.10 g Pr	□ The highest binding affinity, compared to WPH- and EOH-CGA complexes	[104]
SPI	Covalent	Alkaline method	20, 40, 60, 80, 100 μM/g Pr	□ Increased flexibility and antioxidant activity	[105]
Soybean 7s globulin	Covalent	Alkaline method	0.5 mM/100 mg Pr	□ Reduced IgE binding activity and allergenicity	[63]
	Non-covalent (i.e., H-bond and van der Waals)	Mixing in phosphate buffer	0–12 µM (Pr 10 µM)	□ Partial protein unfolding □ Lower surface hydrophobicity	[74]
Zein	Non-covalent (i.e., H-bond and electrostatic)	Mixing in aqueous ethanol solution	5–40 µM (Pr 0.2 mg/mL)	□ Enhanced mechanical properties	[75]
	Covalent	Alkaline method	0.25 mM/0.2 g Pr	□ Enhanced solubility, thermal stability, and antioxidant activity	[60]
WGH	Covalent	Free radical method	5 mM/2 g Pr	□ Improved foaming and emulsifying stability and antioxidant activity	[67]
WG	Covalent	Alkaline method	0.35 mM/10 mg/mL Pr	□ Reduced IgE binding activity and allergenicity □ Improved thermal stability, antioxidant activity and in-vitro digestibility	[107]
RBP	Non-covalent	Ultrasound treatment + mixing in phosphate buffer	0.1 g/100 mL CGA/1 g/100 mL Pr	□ Lower surface hydrophobicity □ Improved emulsifying properties	[110]
RPH	Covalent	Alkaline method	0–0.125/2.5% (*w*/*v*) Pr	□ Enhanced emulsifying activity and oxidative stability	[61]
	Covalent	Alkaline, enzyme, and free radical methods	10 mg (fixed in all methods)	□ Increased emulsifying and antioxidant activities	[17]
SFP	Covalent	Alkaline method	0.005–1 g/0.1 g Pr	□ Lower surface hydrophobicity	[112]
	Covalent and non-covalent	Alkaline or neutral method (pH 9 or 7)	CGA/Pr M ratios of 1:10, 1:5, 1:1, 5:1, and 10:1	□ Improved solubility □ Effect on protein gel strength	[64]
HP	Non-covalent	Ultrasound treatment + mixing	0.1%	□ Improved the structural and functional properties	[113]
QPH	Non-covalent	Mixing in phosphate buffer	0, 20, 120, and 240 μM/g Pr	□ Lower surface hydrophobicity □ Synergistic antioxidant effect	[114]
FP	Covalent	Alkaline method	CGA/Pr mass ratios 0.35%, 0.70%, 1.05% and 1.40%	□ Increased WHC and antioxidant activity □ Decreased OHC and hydrophobicity	[115]
PPI	Non-covalent (mainly electrostatic)	Mixing in phosphoric acid buffer	25, 50, 100, 200, and 250 μM/g Pr	□ Enhanced emulsifying and foaming properties and in vitro digestibility	[116]

CGA: chlorogenic acid; Pr: protein; SPH: soy protein hydrolysate; SPI: soy protein isolate; whey protein hydrolysate (WPH), egg ovalbumin hydrolysate (EOH); WGH: wheat gluten hydrolysate; QPH: quinoa protein hydrolysate FPI: flaxseed protein; PPI: pea protein isolate; HP: hemp protein; WHC: water-holding capacity; OHC: oil-holding capacity; SFP: sunflower protein; RBP: rice bran protein; RPH: rice protein hydrolysate; WG: wheat gliadin.

## 5. Protein–CGA Interactions in Food Systems

Nowadays, the interactions between proteins and polyphenols have become a hot topic in the food and beverage industry in order to develop healthy products with better techno-functional and biological properties. This section aims to overview the potential application of protein–CGA complexes/composites in various food and beverage products.

### 5.1. Beverages

Previous studies highlighted that the bioavailability of polyphenols, e.g., CGAs, may be affected by interactions with dietary compounds, especially proteins, and reported that the simultaneous consumption of coffee and animal or plant milk can significantly affect the absorption of CGA in humans. Furthermore, the potential synergistic effect between proteins and CGAs could open avenues for the development of functional beverages with improved stability, enhanced nutritional profiles, and unique sensory properties.

In a primary study, Petzke et al. [118] explored the impact of CGA on the nutritional quality of β-LG in rats. This study showed that the conjugation of β-LG and CGA moderately reduced the quality of whey proteins, as evidenced by a decrease in true nitrogen digestibility (TND) of about 4% and an increase in fecal nitrogen excretion from 757 to 815 mg. On the other hand, Duarte and Farah [119] found that the simultaneous consumption of milk and coffee may impair the bioavailability of coffee CGA in humans, so that the amount of CGAs and their metabolites recovered after the consumption of combined coffee–milk was consistently lower (40 ± 27%) compared to coffee alone (68 ± 20%). In another study, Felberg et al. [120] investigated the impact of consuming soymilk and coffee together on the urinary excretion of isoflavones (ISOs), CGAs, and their metabolites in healthy adults with normal blood values and no medication or nutritional supplements. The results showed that the combination of soymilk and coffee did not negatively influence the urinary excretion of isoflavones and their metabolites. However, soy proteins and/or other substances present in soymilk bind CGAs, decreasing their absorption in the upper digestive tract up to 42%. The study also discussed the reversible nature of these interactions and emphasized the need for further research on the interaction between CGAs and soy proteins. On the contrary, Alongi et al. [121] found that the addition of milk and high-pressure homogenization (HPH) treatment increased the bioaccessibility of CGAs from nearly 25% to over 50%, promoting CGAs’ micellarization and reducing their susceptibility to degradation during digestion. Also, the incorporation of fat in concentrations of 0.1, 3.6, or 7.1% reduced the susceptibility of CGAs to degradation by promoting their micellarization and complexation with milk proteins, resulting in higher stability. Furthermore, Ji et al. [114] investigated the effects of QPH beverages on the physicochemical and sensory characteristics of coffee. They revealed that the addition of a quinoa beverage masked the unpleasant sensory characteristics of coffee, such as bitterness and astringency, while enhancing smooth mouthfeel and sweetness. The authors also noted that the interactions between QPH and CGA induce structural changes and improve the techno-functional properties of the protein, by unfolding its structure and decreasing surface hydrophobicity. Moreover, the results showed the synergistic antioxidant effect between QPH and CGA, as well as the improved stability of emulsions. These findings suggest the practicability of using polyphenols to improve the functionality and stability of protein beverages and highlight the need for further research to investigate the actual mechanisms of interactions between proteins and phenolic compounds, especially CGAs.

### 5.2. Films and Coatings

Generally, protein-based films and coatings exhibit unique functional and film-forming properties that can act as external barriers to raw food materials, slowing down spoilage and prolonging their shelf-life. However, the overall performance of these films and coatings is much lower than that of plastic materials, which restricts their industrial applications [122]. In this regard, research in recent years has focused on developing different strategies to improve the functionality of these packaging materials. For instance, Fu et al. [101] prepared a novel bioactive coating made of CGA–gelatin for the preservation of fresh seafood. The authors determined the optimal reaction molar ratio of CGA to gelatin to be 4:1 and confirmed the successful complexation of CGA on gelatin using NMR and FTIR spectroscopy. The CGA–gelatin coatings exhibited better antioxidant activity than free CGA. In addition, these coatings showed strong antibacterial activity against a wide range of bacteria, including Staphylococcus aureus, Escherichia coli, Listeria monocytogenes, and Pseudomonas aeruginosa, which are the main spoilage microorganisms in seafood. In another study, Chen et al. [123] evaluated the fabrication, characterization, and application of gelatin–wheat gliadin electrospun films containing CGA for the preservation of grass carp (Ctenopharyngodon idella) fillets. The results showed that CGA was successfully encapsulated in the film and interacted with proteins, as indicated by the morphology and structural changes of the films. The CGA-containing films also exhibited good stability, hydrophobicity, and antioxidant and antibacterial activities, thus significantly prolonging the shelf-life of grass carp fillets. In a further study, Zou et al. [124] investigated the effects of incorporating CGA, GA, and resveratrol (RES) into a gelatin–chitosan–glycerol edible coating on the preservation of fresh beef. This study revealed that CGA has the most significant improvement effect on fresh beef preservation, protecting its color and texture for 3–6 more days compared to uncoated beef. These results indicate the potential applicability of protein–CGA films and coatings in delaying the oxidative degradation of MPs and maintaining their freshness, color, and texture during storage.

### 5.3. Emulsion-Based Delivery Systems

The emulsion-based delivery systems using protein–CGA conjugates/complexes have attracted widespread attention in recent years for the encapsulation, protection, and release of bioactive substances [125]. For instance, Liu et al. [126] investigated the impact of lactoferrin (LF)–CGA and -EGCG conjugates on the structural and physicochemical properties of β-carotene emulsions. The study confirmed the formation of LF–polyphenol conjugates by SDS-PAGE, which caused changes in the structure and nature of LF. Additionally, the LF–polyphenol conjugates effectively enhanced the physical and chemical stability of β-carotene in oil-in-water emulsions against ionic strength, freeze–thaw, and thermal treatments, as well as ultraviolet light exposure. These findings provide valuable insights into the interaction between CGAs and proteins, particularly LF, and their impact on the physicochemical stability of primary and secondary emulsions. In another study, Zhang et al. [127] formulated lycopene-loaded emulsions using covalently modified WPI with CGA and/or high methoxylated pectin (HMP). This study showed that both binary and ternary conjugates increase the stability of the emulsions, with the best protection provided by the ternary WPI-CGA-HMP conjugates. The results also indicated the importance of the interaction between WPI and CGA in improving the physicochemical stability of lycopene in the emulsions, which provides valuable insights into the potential applications of these conjugates in controlled-release delivery systems. In a further study, Wang et al. [108] demonstrated that the addition of CGA to RBP changed the structural and functional properties of the protein, and RBP-CA emulsions exhibited higher stability than those formed using RBP alone. This study also found that the ultrasonic-assisted treatment significantly improved the stability, encapsulation efficiency, and loading capacity of CGAs in the RBP-CGA emulsions.

Overall, these studies highlight the potential application of protein–CGA conjugates to develop food-grade delivery systems, especially in protecting lipophilic bioactive compounds, which can be further enhanced using additional modification techniques, such as using ultrasound treatment or combining them with other macromolecules.

### 5.4. Natural Food Colorants

**Figure 6:**
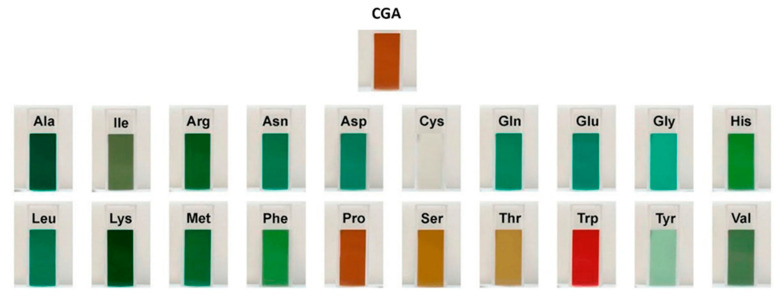
The color of CGA alone and in combination with different amino acids at pH 9.0; adapted from Bongartz et al. [130].

The phenomenon of greening, which occurs during the natural botanical process of some plant products, including sweet potatoes, beets, and sunflower seeds, has attracted considerable attention in the past decades. This phenomenon was attributed to the enzymatic oxidation of CGAs in plant cells and its subsequent reactions with amino acids, such as glycine and lysine, to produce green pigments [128]. Moreover, Liang et al. [129] revealed the positive correlation between the greening intensity of sunflower butter cookies and pH due to the potential effects of acidic conditions on CGA and protein oxidation, as well as their interactions. On the other hand, Bongartz et al. [130] investigated the interactions of CGA with 20 amino acids under alkaline conditions (pH 9.0), which let in the formation of a wide range of colors, including white, green, brown, and, in the case of tryptophan, red (Figure 6). In another study, Moccia et al. [131] developed a new cyanine pigment from the oxidative coupling of CGA with tryptophan with high stability at different pH levels and high temperatures. It also exhibited intense coloring in various food matrices with no observed toxicity on cell lines, making it a promising alternative for red food coloring. These results indicate that the amino acid type and pH level can significantly affect both the nutrition and appearance of the final product. Therefore, understanding the interactions between CGAs and individual amino acids under different environmental conditions may provide valuable insights into developing pH-dependent natural pigments in food and intelligent food packaging systems.

## 6. Research Gaps and Future Perspectives

The study of protein–CGA interactions has made significant progress in recent years; however, several research gaps still remain, providing exciting opportunities for future discoveries to investigate the potential food and non-food applications of these bioactive conjugates and complexes. In this sense, further investigation is necessary to provide the specific binding sites, conformational changes, and thermodynamic parameters involved in protein–CGA interactions at the molecular level to elucidate the precise influence of different types of covalent and/or non-covalent interactions on the structure of proteins and bioavailability of CGAs. In addition, it should be further evaluated how processing conditions, such as pH, temperature, pressure, and ionic strength, affect the protein–CGA interaction, which can help predict the behavior of intra- and extra-molecular interactions of proteins and CGAs during food processing and storage. It is also essential to assess how these interactions affect taste, aroma, and overall consumer acceptability of end-products for their successful applications.

Moreover, while previous studies have focused on specific proteins, comparative investigations of CGA interactions with different types of proteins from various origins can expand their applications in food and non-food matrices. Moreover, despite the promising health benefits associated with CGAs, more studies are needed to understand the actual mechanism of protein–CGA conjugates/complexes in different organ systems and clinical conditions to ensure the well-being and safety of consumers. Also, limited research has investigated the effects of these conjugates and complexes on the gut microbiome. Therefore, future studies could use in vitro models of gut fermentation and metagenomics analysis to assess the impact of these interactions on gut health.

In summary, future research perspectives should aim to determine the exact mechanism of protein–CGA interactions in different conditions, fill existing knowledge gaps in terms of food processing and safety, and discover the innovative applications of protein–CGA conjugates and complexes in food and pharmaceutical products. This may include the development of new food formulations, controlled-release mechanisms, or encapsulation systems.

## 7. Concluding Remarks

This study highlights the importance of interactions between proteins and CGAs as a promising way to improve the techno-functional properties and health-promoting benefits of various proteins from animal and plant sources. CGAs can interact with proteins via covalent and/or non-covalent interactions, each influencing the characteristics of the resulting conjugates or complexes in a different way. The alkaline method is the most widely used technique for the formation of protein–CGA conjugates, which involves the oxidation of CGA to quinones under alkaline conditions (pH 9.0), followed by binding with nucleophilic residues in proteins, mainly through C–S or C–N linkages. On the other hand, hydrogen bonding and hydrophobic forces are the main non-covalent interactions between proteins and CGAs. These interactions alter the secondary and tertiary structures of proteins, subsequently affecting their physicochemical and functional properties, such as solubility, WHC, emulsifying, foaming, digestibility, and gelling characteristics. Notably, the impact of CGAs on these techno-functional attributes of proteins may vary based on the type of proteins and environmental conditions, as well as the CGA concentration. Additionally, the incorporation of CGAs enhances the antioxidant, antimicrobial, antidiabetic, and anticancer activities of proteins, suggesting their potential health benefits in various food formulations and biomedical products.

Overall, these findings underscore the significance of understanding protein–CGA interactions in developing functional foods and pharmaceuticals. However, further studies are needed to assess their functionality in real food products and to understand their gastrointestinal fate using in vitro and in vivo analyses. Moreover, the potential toxicity of these conjugates and complexes should be addressed for their widespread utilization in food and non-food products.
